# Effects of Fibrous By-Products on Growth Performance, Ileal Nutrient Digestibility, Intestinal Morphology, and Microbiota Composition in Weaned Piglets

**DOI:** 10.3390/microorganisms13112482

**Published:** 2025-10-30

**Authors:** Huilin Ouyang, Łukasz Grześkowiak, Wilfried Vahjen, Jürgen Zentek, Beatriz Martínez-Vallespín

**Affiliations:** Institute of Animal Nutrition, Department of Veterinary Medicine, Freie Universität Berlin, Königin-Luise-Str. 49, 14195 Berlin, Germany; h.ouyang@fu-berlin.de (H.O.); juergen.zentek@fu-berlin.de (J.Z.)

**Keywords:** dietary fibre, agricultural by-products, piglets, nutrient digestibility, intestinal morphology, intestinal microbiota, short-chain fatty acids

## Abstract

Three fibrous by-products were evaluated over a 35-day feeding period in 64 weaned piglets, randomly assigned to four groups: a control without by-products (CON) and three others with diets containing 8% carrot pomace (CRT), 8% brewers’ spent grain (BSG), or 8% carob pods (CRB). The growth performance, feed intake, feed conversion ratio, and apparent ileal digestibility of protein and amino acids were not affected. The jejunal and colonic morphology showed no statistical differences, although small numerical increases in the villus height and villus height-to-crypt ratio were noted with the by-products. Total short-chain fatty acid concentrations were stable, but their profile shifted: acetate increased in CRT and CRB (*p* < 0.001) mainly at the expense of propionate (*p* = 0.005). The microbiota composition in the proximal colon showed modest changes, with the highest *Bifidobacterium* spp. abundance in CRT and lowest in CRB (*p* = 0.042), reduced *Ruminococcaceae* UCG 005 with all the by-products (*p* = 0.008), and greater microbial richness in CRB (*p* = 0.009). These results suggest that a moderate inclusion of fibrous by-products may influence intestinal microbial ecology and fermentation patterns without negatively affecting performance or nutrient digestibility in weaned piglets, with no source appearing superior, thereby highlighting their potential as sustainable feed ingredients.

## 1. Introduction

Dietary fibre (DF) has gained prominence in pig nutrition due to its potential to promote gut health and modulate digestive physiology. It is noteworthy that many swine nutrient requirement tables still lack formal fibre recommendations. Traditionally, DF has been restricted in concentrate-based diets for growing pigs because it is often associated with reduced energy and protein digestibility, ultimately impairing animal performance [1,2,3,4]. Nevertheless, the impact of DF on production performance remains controversial and is influenced by the type and characteristics of the fibre used.

The definition of fibre itself is also challenging due to the diversity of fibre compositions and the variety of analytical methods available [1]. DF primarily consists of non-starch polysaccharides (NSP) and lignin, although it may also include minor associated components such as proteins and phenolic compounds [5]. The NSP cannot be digested by the endogenous digestive enzymes, and most of them enter the large intestine serving as a nutrient source for the intestinal microbiota. However, the different composition of the fibre fraction of the feed materials may affect the degree of bacterial fermentation [6]. An increase in fibre seems to have an effect on intestinal differentiation with a higher cell turnover of the intestinal mucosa, although the magnitude of the changes depends on several factors such as the physico-chemical characteristics of the fibre or physiological parameters [7]. Information on the immunomodulatory effects of fibre in pigs remains limited. It is well accepted that microbial fermentation of fibre into short-chain fatty acids (SCFA) can play a role in shaping immune responses [8,9,10].

The increasing demand for antibiotic alternatives in livestock production, especially for managing enteric disorders, has underscored the potential of dietary fibre as a functional feed component. In this context, several reviews have compiled current knowledge to clarify the effects of dietary fibre on digestive physiology and gut health [4,5,11]. Although research results continue to emerge, many issues remain unresolved.

In addition to its physiological relevance, the use of regional by-products obtained in different industrial processes as ingredients for animal nutrition is an interesting strategy in terms of economy, reducing the dependence on imported ingredients as well as the competition with the food industry. It is also highly relevant to sustainability, as it promotes the recycling of these industrial by-products, reduces emissions linked to the long-distance transport of imported feed, helps prevent biodiversity loss induced by the increase in agricultural land used for feed ingredients, and lowers overall environmental costs [12,13,14,15].

The current study aimed to evaluate the effects of three different fibrous by-products (carrot pomace, brewers’ spent grain, and carob pods) when included at 8% in the diet of weaned piglets. The outcomes assessed included growth performance, apparent ileal digestibility of protein and amino acids, intestinal barrier function, and gut microbiota, in comparison to a standard control diet. We hypothesised that the distinct chemical compositions of the by-products would differentially influence digestive physiology and the intestinal microbiota.

## 2. Materials and Methods

### 2.1. Animals, Diets, and Sampling

A total of 64 purebred Landrace piglets were weaned at 27 ± 1 days of age, with an average body weight (BW) of 7.14 ± 0.11 kg, and randomly distributed into four experimental groups, balancing for sex and BW. Animals were allocated to pens (*n* = 2 animals/pen) with ad libitum access to feed and water. The room temperature was set to 29 °C at weaning, and it was decreased until it reached 22 °C at the end of the experimental period. A lighting regimen of 16 h light and 8 h darkness was maintained throughout the experiment.

A control diet (CON) was formulated to meet or exceed the nutrient requirements of weaning pigs according to the recommendations of the Society of Nutrition Physiology [16]. Three fibre-enriched diets were formulated by replacing part of the corn and soybean meal with 8% of either carrot pomace (CRT), brewers’ spent grain (BSG), or carob pods (CRB). To account for differences in the physical form of the by-products, they were ground to obtain a uniform particle size (Table A1). All the diets included 0.4% of titanium dioxide (TiO_2_) for determination of apparent ileal digestibility (AID). The composition of the diets is shown in Table 1.

At 35 and 36 days, eight animals/group were sedated with 20 mg/kg BW of ketamine hydrochloride (Ursotamin; Serumwerk Bernburg AG, Bernburg, Germany) and 2 mg/kg BW of azaperone (Stresnil; Jansen-Cilag, Neuss, Germany) and euthanised by intracardial injection of 10 mg/kg BW of a mixture of tetracaine hydrochloride, mebezonium iodide, and embutramide (T61; Intervet, Unterschleißheim, Germany).

Immediately after euthanasia, digesta samples from the ileum and proximal colon were collected. The samples were placed in sterile tubes, snap-frozen in liquid nitrogen, and stored at −80 °C until analysis. Additionally, small sections of the jejunum and colon were collected for histomorphological studies.

### 2.2. Growth Performance

Individual BW was recorded at weaning (day 0) and thereafter until the end of the experimental period (day 35). Average daily gain (ADG) was calculated based on changes in BW over time. Feed intake (FI) was also monitored and used to calculate average daily FI. Finally, the feed conversion ratio (FCR) was determined as the ratio of daily FI to ADG.

### 2.3. Chemical Analyses and Nutrient Digestibility

Chemical analyses of feed or digesta samples included Weende constituents and, additionally, amino acids (AA), calcium (Ca), phosphorus (P), and total dietary fibre (TDF). Analyses were performed in accordance with the methods issued by the Association of German Agricultural Analytic and Research Institutes [17]: dry matter: VDLUFA III 3.1; crude protein (CP): VDLUFA III 4.1.2 modified according to macro-N determination (vario MAX CN); AA: VDLUFA III 4.11.1; crude ash: VDLUFA III 8.1; ether extract: VDLUFA III 5.1.1; and Ca and P: VDLUFA VII 2.2.2.6. Amylase-treated neutral detergent fibre (NDF) and acid detergent fibre (ADF) were analysed according to VDLUFA (6.5.1 and 6.5.2, respectively) with an automated fibre analyser (ANKOM 2000 Automated Fiber Analyzer, Macedon, NY, USA) using filter bags instead of filter crucibles, and the results were corrected for ash. Total dietary fibre (TDF), insoluble dietary fibre (IDF), and soluble dietary fibre (SDF) were analysed according to the protocol K-TDFR-100A/K-TDFR-200A 08/16 Megazyme (Wicklow, Ireland). Analyses of Ca and P were performed by atomic absorption spectrometry in an AA vario 6 spectrometer (Analytik Jena AG, Jena, Germany), as previously described [18]. The AA analyses were performed on a Biochrom 30 analyser (Biochrom Ltd., Cambridge, UK) after hydrolysis of lyophilised samples in 6 M aqueous HCl at 110 °C for 24 h. Methionine and cystine were measured after oxidation (H_2_O_2_/formic acid).

Analysis of SCFA was carried out by gas chromatography on an Agilent 6890N with a flame ionisation detector, auto sampler G2614A, and auto injector G2613A (Agilent Technologies, Santa Clara, CA, USA). An Agilent 19095N-123, HP-INNOWAX polyethylene glycol column was used for the separation of the SCFA. For sample preparation, 0.5 g of digesta was diluted with 1.0 mL of ice-cold 100 mM 3-(N-morpholino) propanesulfonic acid buffer (pH 7.5), homogenised for 1 min, and incubated for 10 min on ice. Samples were then homogenised again and centrifuged at 17,000× *g* at 4 °C for 10 min. Supernatants were kept on ice, and 100 μL was used for SCFA analysis. Samples were centrifuged, and the supernatants were filtered by a 0.45 μm cellulose acetate syringe filter.

Pooled ileal samples were ground to pass through a 0.25 mm screen and analysed for CP and AA. Nutrient digestibility was calculated based on TiO_2_ as an inert marker, using the method described by Short et al. [19] according to the following formula:

$$AID\;(\%)=100-\left(\frac{\%\;marker\;in\;feed}{\%\;marker\;in\;ileum}\times \frac{\%\;nutrient\;in\;ileum}{\%\;nutrient\;in\;feed}\right)\times 100$$

### 2.4. Histological Analyses

Jejunum and colon sections were fixed in 4% phosphate-buffered formaldehyde (Carl Roth GmbH, Karlsruhe, Germany), dehydrated in increasing concentrations of ethanol (from 70% to 96%) and isopropanol, and cleaned with xylol. Thereafter, the samples were immediately embedded in paraffin wax. Then, 5 µm sections were cut with a sledge microtome (Type 1400, Leitz, Wetzlar, Germany), mounted on glass slides, and dried in an incubator at 37 °C. Prior to staining, tissue sections were deparaffinised in xylene and rehydrated through a descending ethanol series.

For morphometric measurements, haematoxylin/eosin staining was performed, allowing for the measurement of the villus height (VH) in the jejunum and crypt depth (CD) in the jejunum and colon using a light microscope (Photomicroscope III, Zeiss, Germany) equipped with a digital camera (DP72, Olympus, Hamburg, Germany). In colon samples, Alcian blue pH 2.5–periodic acid–Schiff staining was also carried out to count goblet cells (GCs) and to distinguish acidic, neutral, and mixed acidic/neutral mucins, as described previously [18]. The number of GCs was expressed per 1 mm of the basement membrane length.

### 2.5. Determination of Colonic Microbiota

Samples from the proximal colon digesta (200 mg) were subjected to total genomic DNA extraction using a commercial kit (Qiagen Stool kit, Qiagen, Hilden, Germany), according to the manufacturer’s instructions.

The extraction protocols were preceded by repeated bead-beating on a FastPrep-24™ 5G homogeniser (MP Biomedicals, LLC, Santa Ana, CA, USA) to increase DNA extraction efficiency from spore-forming and likely also Gram-positive bacteria [20]. The DNA concentration was determined using the QuantiFluor^®^ dsDNA System (Promega, Walldorf, Germany), following the manufacturer’s instructions. The extracted DNA was sequenced by LGC Genomics GmbH (Berlin, Germany) on a MiSeq device (San Diego, CA, USA) (LGC Inc., 2015). Exact amplicon sequence variants (ASVs) and their respective abundances in each sample were identified using DADA2. Taxonomic classification was performed with QIIME2’s feature classifier in combination with the SILVA SSU database (release 132). A detailed description of the post-sequencing workflow is available in Grześkowiak et al. [21].

### 2.6. Statistical Analysis

Data were analysed using the program SPSS Statistics (Version 25; IBM, Somers, NY, USA). The normality of the data was assessed using the Shapiro–Wilk test. Data that followed a normal distribution were subjected to one-way analysis of variance (ANOVA), with the group as the independent variable, followed by Tukey’s post hoc test for pairwise comparisons. For non-normally distributed data, the non-parametric Kruskal–Wallis test was used. The homogeneity of variances was tested using Levene’s test. The animal served as the experimental unit for most parameters measured, except for feed intake, where the box was considered the experimental unit. Differences at *p* ≤ 0.05 were considered significant.

## 3. Results

### 3.1. Growth Performance and Apparent Ileal Digestibility

All groups exhibited steady BW gains with no significant differences throughout the trial (Table 2). Although the ADG varied numerically, these differences were not significant. Over the entire period, the CRB group had the highest numerical ADG (337 g/day), while the CON had the lowest (286 g/day). Daily FI did not differ significantly among groups, although CRB had a numerically higher intake, exceeding the CON, CRT, and BSG by 21.1%, 14.2%, and 14.7%, respectively. The FCR did not differ significantly across treatments.

The inclusion of the different by-products had no effect on the apparent ileal digestibility (AID) of crude protein (CP) or amino acids (AA) with the exception of the AID of serine which was lower in the CRB group (*p* = 0.002; Table 3).

### 3.2. Histomorphology

None of the measured parameters differed significantly between treatment groups (Table 4). However, in the jejunum, some numerical differences were observed, with the VH ranging from 426 μm (CON) to 575 μm (CRB) and the CD ranging from 318 μm (BSG) to 409 μm (CON). Similarly, the VH-to-CD ratio (V/C) was numerically higher in the BSG and CRB groups compared to the CON, but these differences were not significant.

Goblet cell profiling showed a predominance of acidic goblet cells across all groups, followed by mixed GCs and a low number of neutral GCs. Although there were some numerical differences among the groups, particularly in acidic GCs, no statistically significant differences were observed for either the type of mucin or for the total cell count.

### 3.3. Microbial Metabolites

Total SCFA concentrations in the proximal colon were unaffected by the dietary fibre source (Table 5); however, the SCFA profile was significantly altered. Acetate proportions were lower in the CON and BSG group (*p* < 0.001), while propionate was highest in BSG and lowest in CRT and CRB (*p* = 0.005).

### 3.4. Microbial Composition and Diversity

The microbial abundance of the dominant bacterial taxa in the proximal colon showed limited variation among the groups (Table 6). Overall, taxa of *Clostridium* sensu stricto 1, *Lactobacillus*, and *Terrisporobacter* dominated the colonic microbiota of the study animals. The abundance of *Ruminococcaceae* UCG 005 was the highest in the CON as compared to the CRT and CRB groups (*p* = 0.008). *Bifidobacterium* spp. showed the highest abundance in the CRT group, while their abundance was the lowest in the CRB group (*p* = 0.042).

There was a trend for a higher abundance of the *Coprococcus* 3 taxon in the CRT group (*p* = 0.063), *Dialister* and *Olsenella* in the CRB group (*p* = 0.075 and *p* = 0.065, respectively), and *Ruminococcaceae* UCG 008 in the CRT group (*p* = 0.057).

The richness index was the highest in the CRB group, followed by BSG, CRT, and the CON (*p* = 0.009). The Shannon and evenness indices did not differ among the study groups.

## 4. Discussion

The current experiment used a moderate inclusion of three fibre-rich materials in the feed of newly weaned piglets. The three ingredients were chosen for their markedly different fibre compositions. Carrot pomace is a by-product obtained after the extraction of carrot juice, and its composition is very variable, depending mainly on the variety but also on the processing methods [22]. The main component of the DF fraction included in the pomace is cellulose, but it also contains pectins [23]. Brewers’ spent grain is the main by-product of the brewing industry. It is a lignocellulosic material composed mainly of cellulose, hemicellulose (primarily arabinoxylan), and lignin, containing appreciable amounts of monosaccharides such as glucose, xylose, and arabinose, along with a considerable protein content [24]. This by-product is highly heterogeneous, and its composition depends on various factors related not only to the cereal used (e.g., variety, time of harvesting) but also to the brewing process itself [25]. Finally, the carob tree is a Mediterranean species from the Fabaceae family that has traditionally been used in both human and animal nutrition. Following the extraction of gums from the seeds and sugars from the pods, the residual material is characterised by a high content of insoluble fibre with high levels of polyphenols, mainly tannins [26].

The inclusion level of 8% was selected as a moderate and literature-supported dosage, considered safe for weaned piglets to avoid anti-nutritional effects while allowing measurable responses [27,28,29]. Supplementing the diet of weaned piglets with 8% of any of the tested by-products did not influence growth performance, regardless of the fibre content or composition. This finding aligns with previous studies showing that supplementing piglet diets with low or moderate concentrations of different fibrous by-products does not impair growth performance traits [30,31,32,33]. However, the use of DF has been often linked to impaired performance as a consequence of a decrease in energy and protein digestibility [34,35]. The reduced digestibility associated with DF may be attributed to factors such as an increased intestinal passage rate, higher viscosity of the digesta, and binding of nutrients. Nevertheless, the extent of these effects appears to depend on various factors, including the type of fibre and its particle size. In the current study, the three by-products initially differed significantly in particle size; thus, they were ground to minimise this effect in subsequent comparisons. This could also explain why the AID of CP was similar among them and to that of the control group.

In the case of the AID of serine, which was drastically low in CRB as compared to BSG, CRT, and the CON, the finding is difficult to interpret. Some analytical artefacts such as incomplete hydrolysis or interference during chromatography may explain this low value. To our knowledge, no fibre-based study to date has reported specific effects on serine, supporting the interpretation of an analytical artefact rather than a biological response.

The absence of notable alterations in the intestinal histomorphology suggests that the increase in DF did not compromise gut structure, which is further corroborated by the lack of observed effects on animal performance and protein digestibility. Although the VH and V/C did not differ significantly among treatments, numerical trends suggest a potential increase in the absorptive surface area, particularly in the BSG and CRB groups. In line with this observation, previous studies have reported that IDF may increase the jejunal VH [33,36,37]. On the other hand, different studies have consistently reported that SDF exerts a negative effect on the absorptive surface, mainly through reductions in the VH or V/C [32,38]. This adverse effect is generally attributed to the increased viscosity of the intestinal contents induced by SDF [37]. Nonetheless, in the present study, no detrimental effects on the jejunal epithelial morphology were observed despite the higher SDF content in the CRT group.

Regarding the mucosal barrier, DF seems to influence mucin production differently, depending on several factors, including its solubility, its mechanical impact on the intestinal mucosa, the specific region of the gut affected or the fermentability of the fibre, and the resulting production of SCFA [39]. Thus, SDF has been suggested to have a role in the maintenance of the mucosal barrier by both promoting the proliferation of goblet cells, responsible for mucin production, and increasing the expression of mucin genes, although pectin may be an exception [40,41,42]. Consistent with the results observed in the jejunum, the current study showed no effect on the colonic epithelial morphology or goblet cell counts. It should be noted that the three by-products were included at moderate levels, which may not have been sufficient to produce detectable effects on the intestinal morphology.

The digestibility rate of DF in the small intestine is very low, with an average of 0.200, and depends mainly on the NSP present [43]. As a result, most of the DF remains unfermented and reaches the large intestine, where the longer transit time allows intestinal bacteria to ferment it more intensively. Components of the soluble fibre fraction, including pectins, β-glucans, and fructans, are generally fermented extensively in the large intestine, while hemicellulose and cellulose exhibit greater resistance to bacterial degradation. However, the degradability of cellulose, arabinoxylans, and xylans is variable and influenced by numerous factors, including particle size, transit time, and their bond to other components [43,44]. The microbial fermentation of DF produces SCFA, among them butyrate, which serves as a key energy source for intestinal epithelial cells, supports gut health by enhancing mucosal barrier integrity, and modulates immune responses against inflammation [11,45,46]. In the current study, the SCFA profile was affected by the three tested by-products. Previous studies have also shown that variations in DF characteristics can affect the SCFA composition [11]. The higher amount of soluble NSP present in carrot pomace may have promoted the growth of acetate-producing bacteria such as *Lactobacillus* spp., *Bifidobacterium* spp., or *Bacteroides* spp. In contrast, due to processing methods, the proteins in brewers’ spent grain may be of lower quality and may have reduced ileal digestibility [47], allowing a slightly higher amount of protein to reach the large intestine. This may have stimulated propionate-producing bacteria such as *Prevotella* spp., *Clostridium* spp., or *Veillonella* spp., which could have hindered the potential effects of the DF on increasing acetate levels, thereby resulting in a similar SCFA profile to that of the CON.

In the present study, the majority of the dominant bacterial taxa identified in the proximal colon belonged to the phylum Firmicutes, predominantly within the class *Clostridia*, consistent with previous findings [48]. A study monitoring the evolution of the faecal microbiota in piglets from birth to nine weeks of age found that, at the last time point, *Clostridium* sensu stricto 1, *Lactobacillus*, and *Terrisporobacter* were among the most abundant taxa [21]. In our study, the *Lachnospiraceae* family also exhibited particularly high prevalence. The abrupt transition from lactation to solid feeding, as commonly experienced by piglets under practical farming conditions, is a major driver of profound shifts in the gut microbiota composition. While several bacterial families show marked changes in relative abundance during this transition, the *Lachnospiraceae* family appears to remain comparatively stable [49]. Regarding the role of DF in the abundance registered in the current study, only slight significant changes were observed, although some numerical differences can be seen in some taxa. Surprisingly, *Ruminococcaceae* UCG 005 were most abundant in the CON, which is unexpected given their known role as efficient fibre fermenters and SCFA producers [50,51]. This observation may reflect a compensatory response within the microbial community, potentially balancing shifts in other taxa or metabolic functions. *Bifidobacterium* spp. exhibited the highest abundance in the carrot pomace group, likely reflecting their preference for soluble, readily fermentable substrates compared to insoluble fibres [52].

It is important to note that a higher relative abundance of specific taxa does not necessarily reflect increased metabolic activity and should be interpreted with caution. Some bacteria may be abundant yet metabolically inactive or slow growing under certain conditions. As RNA-based sequencing, which could confirm active metabolism, was not performed, our interpretations are limited to predicted metabolic potential based on taxonomic profiles and known functional traits.

Although no clear conclusions can be drawn from the microbial abundance results, a strong dietary effect was observed in the richness index, which increased in the CRB group, indicating a higher number of distinct microbial taxa. DF is known to influence gut microbial diversity [53]; however, this effect may depend on the type of fibre or the ratio between soluble and insoluble fractions [54]. In our case, a higher IDF content appears to be associated with greater richness, in contrast to other studies in pigs reporting increased microbial diversity driven by higher SDF levels [55]. Conversely, a recent study in mice supports our findings [56].

The heterogeneous and chemically complex nature of the DF fraction makes it difficult to elucidate its specific physiological effects. Variability in fibre sources, degree of polymerisation, solubility, and fermentability further complicates attribution of observed microbial or metabolic outcomes to individual fibre components or their interactions.

## 5. Conclusions

In conclusion, moderate levels (8%) of carrot pomace, brewers’ spent grain, or carob pods in weaned piglet diets did not impair growth performance, nutrient digestibility, or intestinal morphology. Modest shifts in colonic SCFA profiles and microbial richness suggest that these by-products may influence microbial fermentation patterns. Nevertheless, no source appeared superior, and the functional implications of these changes remain unclear. Given their nutritional value and availability as agro-industrial residues, these ingredients could represent promising components in sustainable pig nutrition strategies. Further research with higher inclusion levels, longer feeding periods, and functional microbiome analyses is warranted to clarify their long-term impacts on gut microbial activity, host physiology, and overall production efficiency.

## Figures and Tables

**Table 1:** Composition of experimental diets and their analysed nutrient content (as-fed basis).

	CON	CRT	BSG	CRB
Ingredients (%)				
Corn	30.3	16.2	23.6	16.6
Soybean meal	22.3	23.7	18.7	23.5
Barley	20.0	20.0	20.0	20.0
Wheat	20.0	20.0	20.0	20.0
Soybean oil	2.14	6.72	3.97	6.54
Monocalcium phosphate	1.45	1.58	1.65	1.60
Calcium carbonate	1.24	1.20	1.19	1.17
Vitamin–mineral premix ^1^	1.20	1.20	1.20	1.20
L-Lysine HCL	0.54	0.54	0.67	0.52
DL-Methionine	0.19	0.22	0.27	0.22
L-Threonine	0.17	0.19	0.27	0.19
L-Tryptophan	0.05	0.05	0.08	0.05
Titanium dioxide	0.40	0.40	0.40	0.40
Carrot pomace	-	8.00	-	-
Brewers’ spent grain	-	-	8.00	-
Carob pods	-	-	-	8.00
Analysed composition (g/kg)				
Dry matter	898	906	900	903
Starch	424	351	388	346
Crude fat	40.5	78.9	65.5	76.2
Crude protein	207	208	209	207
Crude ash	53.8	56.1	56.2	56.0
Crude fibre	31.5	49.2	43.9	43.3
Neutral detergent fibre	119	155	148	147
Acid detergent fibre	38.2	58.9	51.7	90.0
Lignin	5.45	5.03	8.65	36.5
Total dietary fibre	129	173	158	181
Insoluble dietary fibre	125	149	152	177
Soluble dietary fibre	4.92	24.4	5.99	4.38
Calcium	7.76	7.64	8.15	8.01
Phosphorus	6.73	6.71	7.51	6.83

^1^ Contents per kg: 600,000 I.U. Vit. A (retinyl-acetate); 120,000 I.U. Vit. D_3_; 6000 I.U. Vit. E (α-tocopherol acetate); 200 mg Vit. K_3_ (MSB); 250 mg Vit. B_1_ (mononitrate); 420 mg Vit. B_2_ (cryst. riboflavin); 300 mg Vit. B_6_ (pyridoxin-HCl); 1500 μg Vit. B_12_; 3000 mg niacin (niacinamide); 12,500 μg biotin (commercial, feed grade); 100 mg folic acid (cryst., commercial, feed grade); 1000 mg pantothenic acid (Ca d-pantothenate); 60,000 mg choline (chloride); 5000 mg iron (iron carbonate); 5000 mg zinc (zinc sulfate); 6000 mg manganese (manganous oxide); 1000 mg copper (copper oxide); 45 mg iodine (calcium-iodate); 20 mg selenium (sodium-selenite); 140 g sodium (NaCl); 55 g magnesium (magnesium sulfate); carrier: calcium carbonate (calcium min 38%).

**Table 2:** Growth performance of weaned piglets fed diets with different fibre sources (days 0–35).

	CON ^1^	CRT	BSG	CRB	SEM ^2^	*p*-Value
Body weight (kg)						
Weaning	7.12	7.12	7.20	7.13	0.16	0.997
Day 35	15.1	16.2	15.0	16.7	0.46	0.520
Average daily gain (g)	286	319	307	337	11.8	0.491
Daily feed intake (g)	406	430	428	491	15.0	0.205
Feed conversion ratio	1.43	1.37	1.40	1.47	0.02	0.344

^1^ CON = control group; CRT = carrot pomace; BSG = brewers’ spent grain; CRB = carob pods. ^2^ Standard error of the mean.

**Table 3:** Coefficients of apparent ileal nutrient digestibility in weaned piglets fed diets with different fibre sources.

	CON ^1^	CRT	BSG	CRB	SEM ^2^	*p*-Value
Crude protein	0.675	0.661	0.607	0.621	0.016	0.357
Alanine	0.613	0.618	0.591	0.619	0.021	0.969
Arginine	0.735	0.767	0.756	0.771	0.015	0.830
Asparagine	0.680	0.638	0.635	0.687	0.019	0.700
Cystine	0.568	0.561	0.561	0.598	0.025	0.954
Glutamic acid	0.703	0.730	0.742	0.765	0.018	0.668
Glycine	0.352	0.442	0.354	0.459	0.036	0.633
Histidine	0.680	0.684	0.692	0.692	0.015	0.991
Isoleucine	0.634	0.642	0.670	0.670	0.020	0.888
Leucine	0.707	0.709	0.691	0.715	0.017	0.973
Lysine	0.726	0.756	0.797	0.777	0.020	0.635
Methionine	0.875	0.899	0.862	0.860	0.008	0.396
Phenylalanine	0.737	0.730	0.732	0.738	0.014	0.997
Proline	0.670	0.674	0.691	0.704	0.019	0.920
Serine	0.692 ^a^	0.683 ^a^	0.686 ^a^	0.491 ^b^	0.024	0.002
Threonine	0.698	0.715	0.729	0.727	0.015	0.876
Tyrosine	0.727	0.679	0.716	0.732	0.017	0.713
Valine	0.595	0.553	0.615	0.625	0.021	0.672

^1^ CON = control group; CRT = carrot pomace; BSG = brewers’ spent grain; CRB = carob pods. ^2^ Standard error of the mean. ^a,b^ Means with different superscripts within the same row differ significantly.

**Table 4:** Morphometric and goblet cell count in the jejunum and proximal colon of weaned piglets fed diets with different fibre sources.

	CON ^1^	CRT	BSG	CRB	SEM ^2^	*p*-Value
Jejunum						
Villus height (μm)	426	473	486	575	22.9	0.224
Crypt depth (μm)	409	358	318	384	14.7	0.127
V/C ratio ^3^	1.13	1.33	1.59	1.53	0.09	0.217
Proximal colon						
Crypt depth (μm)	356	325	341	334	8.49	0.645
Acidic goblet cells ^4^	55.2	47.7	55.9	52.3	2.86	0.764
Neutral goblet cells	2.94	2.09	0.40	4.07	0.79	0.305
Mixed goblet cells	11.0	14.5	9.02	13.5	2.16	0.820
Total goblet cells	69.1	72.4	66.70	69.9	1.63	0.708

^1^ CON = control group; CRT = carrot pomace; BSG = brewers’ spent grain; CRB = carob pods. ^2^ Standard error of the mean. ^3^ V/C ratio: villus height-to-crypt depth ratio. ^4^ Expressed per 1 mm of basement membrane length.

**Table 5:** Short-chain fatty acid profiles in the proximal colon of weaned piglets fed diets with different fibre sources.

		CON ^1^	CRT	BSG	CRB	SEM ^2^	*p*-Value
Total SCFA	(µmol/g)	86.6	86.5	87.4	83.6	3.04	0.975
Acetate	%	54.0 ^b^	60.2 ^a^	53.0 ^b^	60.2 ^a^	0.89	<0.001
Propionate	%	30.3 ^ab^	26.5 ^b^	31.4 ^a^	25.9 ^b^	0.70	0.005
Butyrate	%	13.0	11.2	13.2	12.0	0.40	0.266
Valerate	%	2.67	2.02	2.42	1.83	0.13	0.084

^1^ CON = control group; CRT = carrot pomace; BSG = brewers’ spent grain; CRB = carob pods. ^2^ Standard error of the mean. ^a,b^ Means with different superscripts within the same row differ significantly.

**Table 6:** Bacterial community composition and diversity in the proximal colon contents of weaned piglets fed diets with different fibre sources.

	CON ^1^	CRT	BSG	CRB	SEM ^2^	*p*-Value
*Clostridium* sensu stricto 1	25.0	17.7	20.4	16.3	2.76	0.928
*Lactobacillus*	19.5	22.8	17.6	19.2	2.19	0.818
*Terrisporobacter*	5.53	8.52	6.68	3.57	0.95	0.563
*Ruminococcaceae* UCG 005	4.24 ^a^	1.54 ^b^	1.83 ^ab^	1.56 ^b^	0.30	0.008
Unknown Family *Lachnospiraceae*	3.11	1.53	1.90	1.95	0.30	0.487
*Catenisphaera*	2.80	2.64	1.60	1.37	0.36	0.674
*Dialister*	2.75	2.66	2.51	5.16	0.35	0.075
*Lachnospiraceae* XPB1014 group	2.72	4.19	2.63	3.15	0.40	0.576
*Christensenellaceae* R 7 group	2.13	3.20	2.18	1.46	0.34	0.495
*Erysipelotrichaceae* UCG 002	2.03	1.19	0.89	1.84	0.25	0.322
Unknown Family *Muribaculaceae*	1.48	0.95	2.04	2.40	0.28	0.254
*Agathobacter*	1.42	0.95	0.41	1.00	0.24	0.159
*Blautia*	1.22	3.48	1.36	0.59	0.56	0.755
*Coprococcus* 3	1.14	7.49	5.99	1.01	0.41	0.063
*Turicibacter*	1.09	0.76	0.95	1.34	0.10	0.211
*Alloprevotella*	0.75	0.64	0.70	0.83	0.06	0.628
*Ruminococcaceae* UCG 002	0.63	0.24	0.17	0.24	0.06	0.172
*Bifidobacterium*	0.61 ^ab^	0.82 ^a^	0.40 ^ab^	0.29 ^b^	0.07	0.042
*Olsenella*	0.60	0.75	1.12	1.67	0.15	0.065
*Ruminococcaceae* UCG 008	0.48	1.87	0.51	1.06	0.20	0.057
*Syntrophococcus*	0.45	0.65	0.44	0.48	0.05	0.284
*Streptococcus*	0.32	1.72	1.56	1.02	0.27	0.326
*Subdoligranulum*	0.25	1.58	1.08	0.83	0.20	0.118
Unknown Family *Ruminococcaceae*	0.24	0.14	0.13	0.10	0.05	0.684
Richness index	158 ^b^	163 ^b^	169 ^ab^	220 ^a^	8.09	0.009
Shannon index	3.39	3.46	3.57	3.93	0.09	0.222
Evenness index	0.67	0.68	0.70	0.73	0.02	0.692

^1^ CON = control group; CRT = carrot pomace; BSG = brewers’ spent grain; CRB = carob pods. ^2^ Standard error of the mean. ^a,b^ Means with different superscripts within the same row differ significantly.

## Data Availability

The original data presented in the study are openly available in Zenodo at 10.5281/zenodo.17465222.

## Appendix A

**Table A1:** Nutritional composition and particle size distribution (after grinding) of carrot pomace, brewers’ spent grain, and carob pods.

	Carrot Pomace	Brewers’ Spent Grain	Carob Pods
Analysed composition (g/kg)			
Dry matter	927	912	909
Starch	202	28.3	54.4
Crude fat	7.01	88.1	6.20
Crude protein	75.5	266	83.4
Crude ash	47.8	54.6	36.5
Crude fibre	181	140	159
Total dietary fibre	623	507	715
Insoluble dietary fibre	428	490	698
Soluble dietary fibre	195	16.1	17.4
Calcium	4.05	6.98	9.57
Phosphorus	1.66	6.57	0.53
Particle size (mm)			
>4.00	0%	0%	0%
4.00–2.50	0%	0%	0%
2.50–1.00	0%	0%	0%
1.00–0.63	1.40%	0.20%	0.20%
0.63–0.40	22.6%	11.1%	14.9%
0.40–0.20	38.2%	42.2%	35.1%
0.20–0.15	11.8%	15.0%	10.5%
0.15–0.10	11.6%	13.3%	10.7%
0.10–0	14.4%	18.2%	28.6%
